# A Case of Pediatric Auricular Erythromelalgia

**DOI:** 10.7759/cureus.21088

**Published:** 2022-01-10

**Authors:** Alyssa Reese, Lauren DiNardo, Kristina Powers, Michele M Carr

**Affiliations:** 1 Otolaryngology, Jacobs School of Medicine and Biomedical Sciences, University at Buffalo, Buffalo, USA

**Keywords:** erythermalgia, red ear syndrome, scn9a, erythromelalgia, pediatric auricular erythromelalgia

## Abstract

Erythromelalgia is a rare clinical syndrome involving erythema, warmth, and burning pain in one or more of the extremities. Primary erythromelalgia is idiopathic and can begin during childhood or adulthood. In the pediatric population, auricular erythromelalgia is rare, and its etiology is not well understood. We present a case of a four-year-old boy who presented with recurrent episodes of red, painful pinnae. We also discuss previous literature on red ear syndrome and erythromelalgia.

## Introduction

Erythromelalgia is a rare clinical syndrome first described in 1878 [1]. The incidence of erythromelalgia is estimated to be 1.3 (0.8-1.7) per 100,000 people per year, as of 2009 [2]. Two forms of erythromelalgia have been reported: primary and secondary. Primary erythromelalgia is idiopathic and can begin during childhood or adulthood [3]. Secondary erythromelalgia presents as a consequence of an underlying disease, typically an autoimmune disorder, myeloproliferative disorder, or neuropathic condition [3,4]. Primary erythromelalgia is more prevalent than secondary with incidences of 1.1 (0.7-1.5) and 0.2 (0.02-0.4) per 100,000 people per year, respectively [4].

This paper discusses the case of a young boy with erythromelalgia of the ears.

## Case presentation

**Figure 1:**
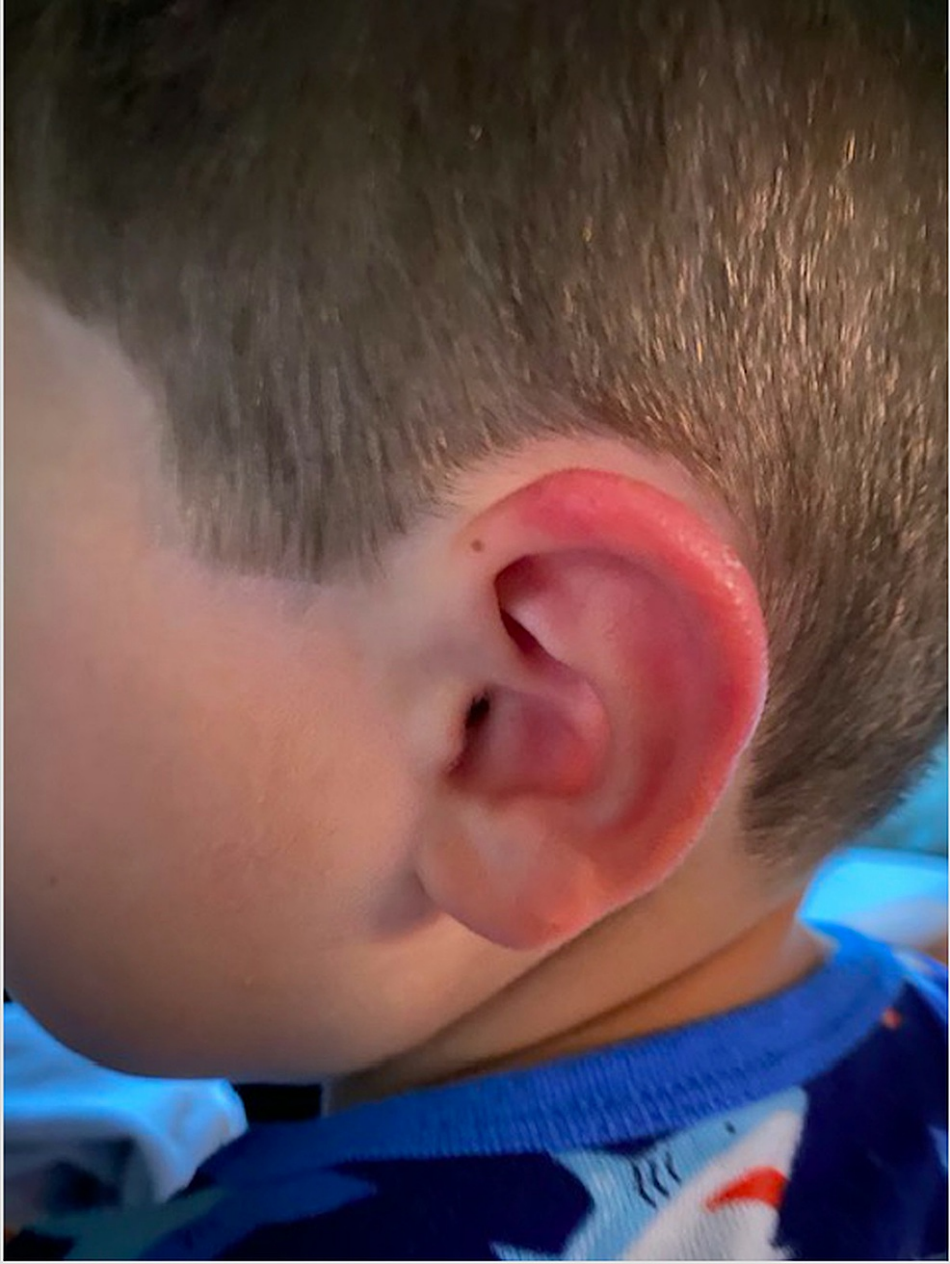
Erythematous left pinna.

A four-year-old boy was seen at a pediatric otolaryngology clinic with the complaint of swollen lymph nodes and redness of the external ears. The patient’s mother reported that the patient initially experienced epistaxis in October 2019, for which he was treated with an antibiotic ointment. Enlarged cervical lymph nodes accompanied the nosebleeds. Following treatment, the epistaxis resolved, but the enlarged cervical lymph nodes continued to present occasionally. During the summer of 2020, approximately eight months following the epistaxis episodes, he began experiencing redness of the pinnae bilaterally, along with the enlarged cervical lymph nodes. The erythema came on gradually, lasted between 20 minutes and several hours, and was associated with burning auricular pain (Figure 1). On two occasions, his mother noticed a blister on his left pinna that took over a week to heal each time. Symptoms occurred at all times of the day with no known triggers. The episodes were occurring more frequently during the winter. Treatment with diphenhydramine did not resolve the redness or pain. Cold packs were placed on the ears for relief, but the child was aggravated by the cold temperature. 

The child was allergic to sunflower extract and had no known drug allergies. He was born at 39 weeks by a planned cesarean section. He was solely breastfed until approximately 3-4 months of age and was then supplemented with bottle feeds until he was completely bottle-fed at six months. He had no other significant prior medical history, hospital stays, or history of middle ear infections. The patient had one older sister who had no significant medical history. His mother had a history of migraine headaches that began in early adulthood, fibromyalgia, and celiac disease. The child’s maternal grandmother also had fibromyalgia, and his maternal great-grandmother had migraine headaches. In addition, his paternal grandmother had a connective tissue disorder. His paternal aunt had celiac disease, and a paternal female cousin had Raynaud’s syndrome and juvenile rheumatoid arthritis.

**Figure 2:**
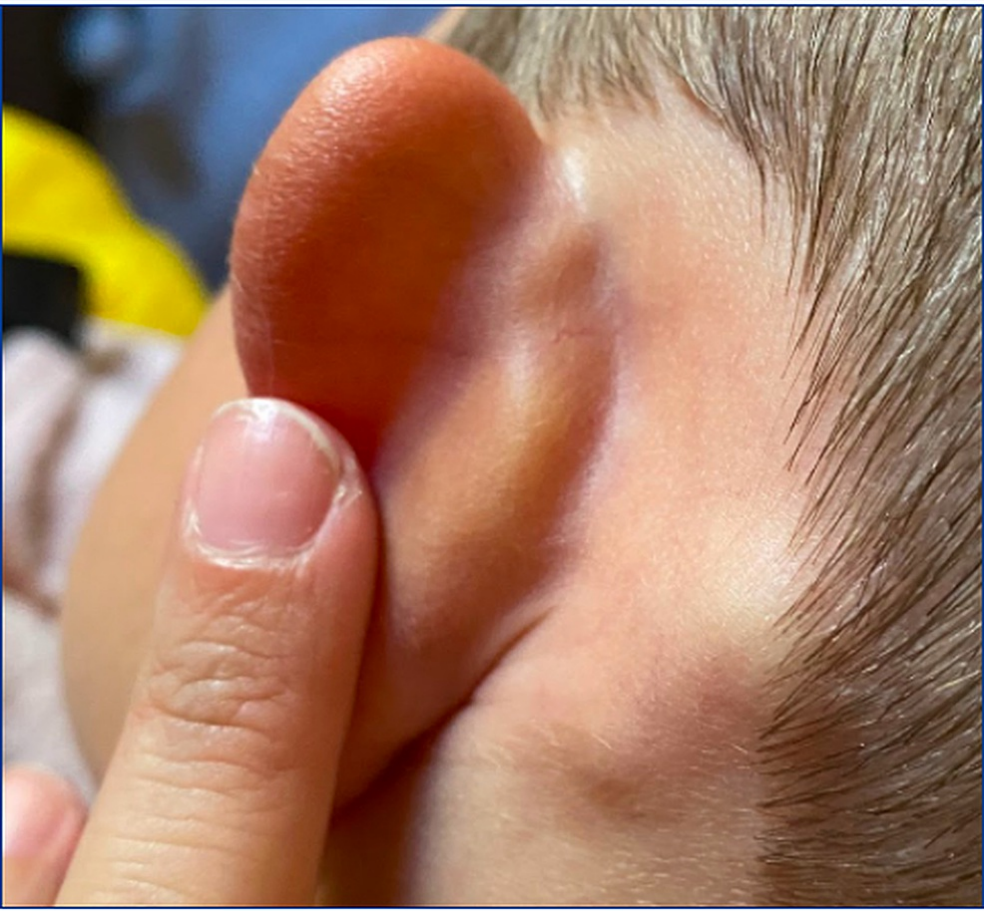
Enlarged left posterior auricular lymph node.

No redness of the pinnae or evidence of chondritis, cellulitis, or swelling was present at the time of examination. The patient had normal tympanic membranes with no evidence of middle ear fluid. The head and neck exam was positive for enlarged cervical lymph nodes, including post-auricular nodes (Figure 2). All were less than 1 cm in diameter, mobile, rubbery, and non-tender. The patient was treated with a two-week course of amoxicillin and returned after two weeks for follow-up. Treatment with amoxicillin resulted in a decrease in the frequency of the episodes but no resolution of the enlarged cervical lymph nodes.

## Discussion

Erythromelalgia is a clinical syndrome characterized by episodic erythema, warmth, and burning pain, typically located on an extremity [4,5]. Symptoms usually present bilaterally and can range from mild to severe. Symptoms are often triggered by warm temperatures or exercise and can be relieved by cool temperature therapy. Relief in pain with cold water is seen in most cases of erythromelalgia, which can be considered pathognomonic [5].

Currently, erythromelalgia is rare and not well described in the pediatric population (Table 1). Some cases of pediatric erythromelalgia have been shown to be inherited and linked to dominant gain-of-function mutations of the SCN9A gene [6]. This gene is linked to voltage-gated sodium channels (V1.1-1.9) located in the heart, nervous system, and muscles [6]. One of the classes of sodium channels (V1.7) is located in the peripheral nociceptive sensory neurons and plays a critical role in sensitivity to pain [6]. A gain-of-function mutation can hyperpolarize the sodium channels, increasing the excitability of the peripheral nociceptors [6]. Understanding this underlying mechanism as a potential cause of erythromelalgia has allowed for testing of more targeted therapies with medications such as lidocaine, mexiletine, and carbamazepine that act on sodium channels [6]. Pediatric erythromelalgia has also been associated with a small-fiber neuropathy based on a case study, an etiology that has been previously described in adults [4].

**Table 1:** Cases of pediatric auricular erythromelalgia in the literature.

	Research Design	Study Population	Relevant Details
Grandy K et al. (2012) [5]	Case report	7-year-old male	The patient had an intermittent, sudden onset of burning sensation and erythema of both of his ears that lasted about 20 minutes. He experienced seven episodes per day that required an ice pack for relief —resolved spontaneously by his 16 monthly follow-up appointment.
Moitri MO et al. (2015) [7]	Case report	5-year-old male	The patient had intermittent erythema of the ears for three years. The symptoms lasted for one hour and resolved spontaneously. Initially, the frequency was once a week but decreased to 2-3 times per month. The patient was diagnosed with red ear syndrome.
Brill TJ et al. (2009) [8]	Case report	7-year-old male	The patient had episodes of reddening, swelling, and a burning sensation in one ear with local hypothermia that persisted for three years. Episodes were more frequent in winter. Differential diagnoses included both red ear syndrome and erythromelalgia.

Erythromelalgia is currently diagnosed based on signs and symptoms of erythema, a burning sensation, increased skin temperature, and associated discomfort [4]. This information is obtained from the history and/or the physical exam, and there is no specific recommended diagnostic testing [4]. It is also predominantly a diagnosis of exclusion as peripheral neuropathy, Raynaud’s phenomenon, vasculitis, Fabry disease, reflex sympathetic dystrophy, and recurrent soft-tissue infections should also be considered [5]. If the erythromelalgia occurs within the pinna, another diagnosis to consider is red ear syndrome (RES), which has a clinical presentation similar to erythromelalgia. RES is characterized by episodes of unilateral erythema, warmth, and burning pain of the ear [7]. Symptoms can be induced by touching the affected area, changes in temperature, or they may arise spontaneously [7]. One of the major distinctions between RES and erythromelalgia is laterality. RES is typically unilateral, while erythromelalgia is bilateral [7]. Additionally, erythromelalgia is worsened with warmth and relieved by cooling, which is not always true with RES [8]. The patient in the present case most likely has an isolated form of erythromelalgia given the bilateral distribution. However, it is important to note that since RES and erythromelalgia have similar diagnostic criteria; it is still unknown if erythromelalgia is a component of RES or if RES is an auricular variant of erythromelalgia [8].

There is no universally effective treatment for erythromelalgia. Most commonly, avoidance of triggers and supportive therapy are advised. Symptoms can be relieved by running cool water over or elevating the affected area [3,5]. Safe cooling options include fans and air conditioning as ice can lead to skin necrosis or ulceration, and prolonged soaking in a water bath can lead to infection [3,5]. The child, in this case, struggled to keep an ice pack on his pinnae, as he found the cooling to be overwhelming. Treatment with medications such as tramadol, gabapentin, prednisone, propranolol, calcium channel blockers, mexiletine, prostaglandin analogs, and IV sodium nitroprusside, and topical agents such as capsaicin and lidocaine also appear in case reports in the literature [9,10]. Invasive approaches such as continuous epidural anesthetic infusions and sympathetic ganglion blocks are other treatment options [10]. Some of these attempted treatments have been shown to have some benefit in a few cases, but none are universally effective. One case study showed that lidocaine patches were effective for pain relief in some pediatric patients [4].

Pediatric erythromelalgia is associated with increased morbidity and mortality. In a case study of 32 children with erythromelalgia, many patients had varying degrees of functional impairment that negatively impacted daily activities. Physical activity was limited in most patients. Twenty-eight percent of these children had anxiety, depression, or behavioral problems, and school attendance was adversely affected in 34%. In this study, three patients died secondary to suicide, sepsis from prolonged soaking of the extremities, and treatment complications [4]. In children, the demographics, natural history, characteristics, etiologies, and treatment options of auricular erythromelalgia are not well understood. This may be due to the limited number of cases reported or recognized. The mean time to diagnose this condition is 5.2 years; this latency may indicate providers' limited understanding of this syndrome and its diagnosis [4]. Another component may be the nature of the disease to cause mild symptoms for which patients do not seek medical attention. It has also been reported that some individuals undergo spontaneous resolution or gradual reduction of the frequency and severity of episodes and may be lost to follow-up [5].

## Conclusions

Currently, erythromelalgia is not well described in the pediatric population and only a few cases of pediatric auricular erythromelalgia exist in the literature. Therefore, recognition, diagnosis, and more frequent reporting of pediatric auricular erythromelalgia are necessary in order to elucidate the etiology and effective treatment.
